# A Modified Customized Rigid Gas Permeable Contact Lens to Improve Visualization During Phacoemulsification in Ectatic Corneas

**Published:** 2019-11-30

**Authors:** Wassef Chanbour, Fadi Harb, Elias Jarade

**Affiliations:** 1 Beirut Eye and ENT Specialist Hospital, Beirut, Lebanon; 2 Mediclinic, Dubai mall, Dubai, UAE; 3 Lebanese University, Beirut, Lebanon

**Keywords:** Customized Rigid Gas Permeable, RGP, Custom Lens, Contact Lens, Phacoemulsification, Keratoconus, Cataract, Nuclear Sclerosis

## Abstract

Advanced Keratoconus and ectatic corneal diseases may lead to corneal thinning and irregular astigmatism. The optical distortion caused by these pathologies may result in poor visibility for the surgeon during phacoemulsification. Thus, the risk of complication would be increased intraoperatively (capsular rupture, vitreous loss). The aim of this case series was to use a modified customized Rigid Gas Permeable (RGP) contact lens to improve visualization during all the stages of phacoemulsification in irregular corneas and to avoid open sky technique for cataract removal during penetrating keratoplasty (PK). A customized, 12 mm, RGP contact lens was designed and manufactured. Two peripheral notches were customized to fit the hand position of the surgeon (at 11 O’clock and 2 Clock in this case series) to allow clear corneal incisions. Six eyes of 6 patients were included (3 eyes with advanced keratoconus and a severely optically distorted, yet clear corneas, planned for PK on the same day; 2 eyes were status post-intracorneal ring segment implantation for stage 4 keratoconus and 1 eye had combined phacoemulsification with superficial keratectomy to remove paracentral corneal Salzmann’s nodule). Lens opacities ranged from +2 to +4 nuclear sclerosis in all eyes. Good visualization of the anterior lens and capsule attained with the RGP contact lens fitting. Improved visualization was reported during all the steps (Capsulorhexis, Irrigation/Aspiration, Phaco. Intraocular lens implantation). The phacoemulsification was smooth and non-complicated in a total of 6 eyes of 6 patients. In these cases, without RGP fitting, the operation was not technically possible. The customized notches allowed any insertion of surgical instruments, and with the help of viscoelastic maintained a good stability of the contact lens during the operation. We concluded that customized method RGP contact lens, may help reducing complications during phacoemulsification in advanced corneal ectasia and perhaps in irregular corneas as well.

## INTRODUCTION

Rigid Gas Permeable (RGP) contact lens has been used to improve the visual acuity in patients with irregular astigmatism. It is used mainly in patients with keratoconus and poor corrected distance visual acuity (CDVA) and to manage the refractive complications after corneal transplantation and refractive surgery [1]. These patients with severe irregular astigmatism can achieve a higher CDVA with RGP contact lenses compared to the spectacle correction [2].While on the cornea, the regular surface of these lenses compensates for the irregular corneal shape and improves the quality of vision. 

Patients with severely distorted corneas may present for cataract surgery or combined cataract and corneal transplant procedures. In severely distorted, yet clear ectatic corneas, surgeon may prefer to do closed system cataract extraction (phacoemulsification) rather than open sky technique to avoid serious complications that are more encountered during open sky cataract extraction technique [3, 4].

In such cases, the usual methods used to avoid corneal dryness and to achieve an optimal visualization of the anterior chamber during all the steps of phacoemulsification include irrigation with balanced salt solution (BSS) or application of viscoelastic agents on the corneal surface [5]. However, BSS may be ineffective in improving surgeon’s visualization in highly irregular corneas and needs continuous application by an assistant. While viscoelastic agents may easily slip off the cornea. Intraoperative use of a RGP contact lens (7.8mm base curve, 8.8 millimeter (mm) diameter, 0 diopter [D] power) has been described during phacoemulsification in patients with severe keratoconus and it was found effective to improve surgeon’s visualization [6]. However, this technique had many drawbacks. Also, RGP lens protected the cornea from drying and provided a better visibility during non-contact vitrectomy [7].

Herewith, we describe a modified technique using a customized RGP contact lens during phacoemulsification to enhance surgeon’s anterior segment field of vision and facilitate surgical manipulation of phaco instruments through clear corneal incision.

## METHODS

This was a prospective case series study approved by the institutional review board in January 2018 at the Beirut Eye and ENT Specialist Hospital, Lebanon. This study complies with the tenets of the Declaration of Helsinki. Patients were recruited over 1-year period. We included those cases with cataract grade 2 or higher and severely optically distorted cornea (keratoconus and irregular astigmatism). However, cases with dense central corneal scar or opacification were excluded. Keratoconus severity was classified according to the classical Amsler-Krumeich classification system [8]. Cataract density was classified using the Lens Opacities Classification System III (LOCS III) [9].

**Lens manufacturing**

**Figure 1:**
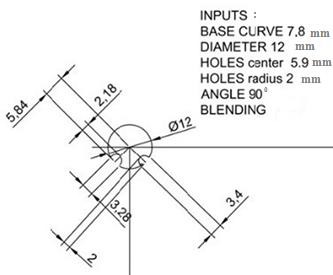
Modified Customized Rigid Gas Permeable Contact Lens Specifications

The RGP lens (Optimum G.P Comfort Extra, Conta Mac, The UK) was customized according to each patient. Base curve was chosen according to the flat keratometry readings. A 12-mm diameter RGP lens was used for total corneal coverage with a lens power of -6.00 D or -9.00D depending on the corneal steepness. Two 60-degree arc length and around 2 mm depth notches were manually fashioned (using a high speed micro drill used in dentistry: air pressure: 240 kilopascal, atomized water pressure: 198 kilopascal, rotation speed: 370000 r/min, burr applicable: 1.6 mm head diameter 12.5mm) on the periphery of the RGP lens at 2 and 10 O’clock at the location of corneal incisions sites (following surgeon preferences) (Figure 1). The lens was disinfected using surgical disinfectant (Anioxide 1000 for 5 minutes) since the gas treatment under 55^0^C may affect the clarity of the lens. The total cost of the lens was 50$.

**Surgical technique**

Phacoemulsification procedures were performed by the same surgeon (E. J.) under topical or local anesthesia. Following surgeon’s preference, a 2.5mm clear corneal incision was done at 10 O’clock and a parenthesis at 2 O’clock. Then the RGP was placed on the cornea over a viscoelastic agent. Capsulorhexis was performed using 27 gauge bent-tip needle and a rhexis forceps. The large diameter of the contact lens allowed a wide view of the anterior lens capsule leading to larger capsulorhexis diameter. Bimanual phacoemulsification was performed while inserting both instruments through the contact lens notches. Cortical lens remnants were removed using the single tipped irrigation/aspiration (I/A) system. Finally, posterior chamber intraocular lens (PCIOL) was inserted and the main wound was secured with one 10-0 nylon suture.°

## RESULTS

**Figure 2:**
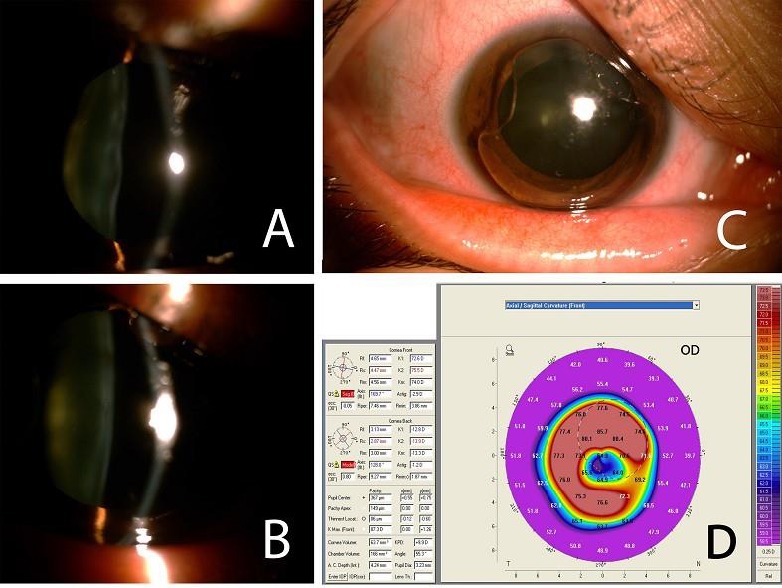
Clinical presentation of patient number 2, A: slit lamp photo using small slit showing irregular anterior capsule surface in the eye without contact lens, B: slit lamp photo after application of rigid gas permeable (RGP) contact lens showing smoothing of anterior capsule, C: slit lamp photo using wide beam showing the notched contact lens in place, D: preoperative topography of the eye showing very severe steepening of the cornea.

Preoperative slit lamp photographs of a patient’s eye with advanced ectasia showed better visibility with smoothing and more regularities of the anterior lens capsule with RGP contact lens fitting compared to non-contact lens fitting, which may allow better visualization of the anterior and most importantly to the posterior capsule as well during the phacoemulsification procedure (Figure 2).

**Figure 3:**
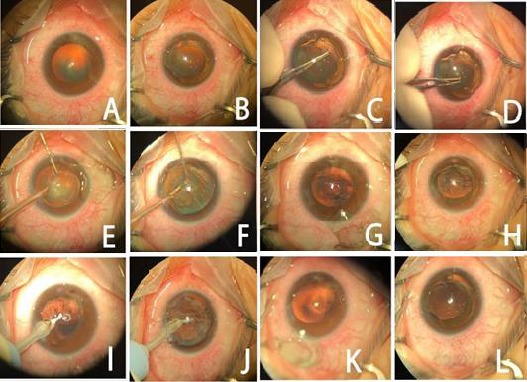
Intraoperative photos of patient number 2 showing optical distortions during all the stages of cataract surgery before and after application of contact lenses respectively: pre-incision: pictures A and B, capsulorhexis C and D (only with application of contact lens), phacoemulsification E and F, after phacoemulsification G and H, irrigation-aspiration I and J, posterior intra-ocular lens implantation K and L.

Non-complicated phacoemulsification was performed using Customized RGP contact lenses in 6 eyes of 6 patients with severely distorted corneas (Table 1). Three eyes had advanced keratoconus with severely optically distorted, yet clear corneas that underwent phacoemulsification followed by keratoplasty; 2 eyes were status post-intracorneal ring segment implantation for stage 4 keratoconus and 1 eye had combined superficial keratectomy to remove paracentral corneal Salzmann’s nodule followed by cataract extraction. Lens opacities ranged from +2 to +4 nuclear sclerosis in all eyes. The contact lens insured a better visualization with a wider view during all the stages of the operation (Figure 3). The lens maintained its stability during ocular manipulation and instruments insertions. Contact lens surface hydration using BSS hydration was not necessary. All procedures of phacoemulsification were performed uneventfully with relatively good visibility mainly for the anterior and posterior capsule. The surgical time was similar to a phacoemulsification in a case with normal corneal anatomy (mean of 5 minutes). Furthermore, instruments manipulation during clear cornel incisions was easily attained. Preoperative and postoperative corrected distance visual acuities of subjects are summarized in Table 1.

**Table 1:** Characteristics of 6 Eyes of 6 Study Subjects Who Underwent Phacoemulsification Using Modified RGP for Intraoperative Visualization.

No	Age (Y)	Diagnosis	Additional procedure	PSH	Sex	KCN Stage	Onset	Kmax	TP	cataract	Eye	SLE	RGP	Pre-op CDVA	Post-op CDVA
1	50	Cataract + KCN	Penetrating keratoplasty	Nil	F	4	25	70.5	242	2+	OS	-Central bulging -inferior thinning	Power: -6 D BC: 6.15 mm	20/200	20/40
2	45	Cataract + KCN (Figure 2 and 3)	Penetrating keratoplasty	Nil	F	4	15	87.3	149	3+	OD	-Fleischer ring - inferior thinning	Power: -9 D BC: 4.7 mm	CF 1.5m	20/30
3	54	Cataract + KCN	Penetrating keratoplasty	Nil	F	4	20	82.1	221	2+	OS	-Fleischer ring -inferior thinning	Power: -9 D BC: 4.9 mm	CF 1.5m	20/40
4	62	Cataract + KCN	Nil	AK ICRS	M	4	25	57.2	473	4+	OS	Irregular surface	Power: -6 D BC: 7.45 mm	20/200	20/60
5	73	Cataract + KCN	(refused penetrating keratoplasty)	ICRS	F	4	22	54	220	2+	OD	-central thinning - irregular surface	Power: -6 D BC: 8.45 mm	20/125	20/60
6	65	Cataract + Salzmann's nodular degeneration	Superficial keratectomy (prior to phacoemulsification)	Nil	M	Nil	Nil	56.6	493	2+	OS	Para-central Salzmann's nodule	Power: -6 D BC: 7.3 mm	20/60	20/30

Abbreviations: No: number; Y: years; PSH: past surgical history; Kmax: maximum keratometric reading in diopter; SLE: slit-lamp exam; KCN: keratoconus; Onset: Age of Onset of KCN; TP: thinnest pachymetry in micrometer; Cataract: Severity Scale and Type of Cataract; Eye: Operated Eye; D: diopter; RGP: rigid gas permeable contact lens Specifications (Diameter 12mm); Pre-op: preoperative; Post-op: postoperative; CDVA: corrected distance visual acuity; F: female; M: male; CF: count finger vision; ICRS: intrastromal corneal ring segment; AK: astigmatic keratotomy; BC: base curve; mm: millimeter; m: meter; OD: right eye; OS: left eye

## DISCUSSION

In this study, RGP contact lenses were used for a safe phacoemulsification in severely optically distorted corneas with better intraoperative visibility, faster performance and a better intraoperative handling of the phacoemulsification instruments through a clear corneal incision. The use of these lenses eliminates the need for an open sky approach to remove cataract during PK and decreased the chances of vitreous loss in 3 cases of keratoconus eyes with severely distorted corneal optic. Moreover, these lenses helped better visualization in phacoemulsification surgery post-intrastromal corneal ring segment implantation in 2 cases and also helped overcome the corneal irregularities after superficial keratectomy in one case. The final CDVA improved in all patients.

RGP contact lenses were chosen over soft lenses because of difficulty in making the peripheral notches at the incision site without breaking the lens. In addition, the inflexible structure of these RGP lenses allows the tears to pool between the lens and the cornea, effectively masking the irregular surface and providing a smooth shape at the corneal plane [10]. These features improve the optical quality of the image soft contact lenses failed to maintain due to its surface irregularities [7].

Intraoperative application of topical viscoelastic has been previously described during phacoemulsification in regular corneas and many prefer it over frequent BSS use, because it provides epithelial protection, surface hydration and optical clarity [11, 12].

Unfortunately, surface viscoelastic presents some drawbacks. Cohesive agents do not provide optimal coating since it forms a sphere that slips over the wet surface, while dispersive agents do not cover surface homogenously and require manual spread resulting in poor visibility [5, 13]. Therefore, RGP contact lens was the best option in our cases.

RGP of larger diameter (12mm) than the one used by Oie, Y et Al [6] provided a larger visual field for the surgeon by covering all the cornea, while maintaining a good stability through the created notches and allowed the surgeon with clear corneal incision preference to perform the operation. Choosing the lens curvature base curve according to the flatter central corneal curvature creates a thinner tear film and eliminates troublesome air bubbles from being trapped inside the central part of the lens [1].

Severe keratoconus with high irregular astigmatism and yet clear corneas is the best candidate for the use of intraoperative lenses. RGP lenses helped to overcome the corneal surface irregularity by providing a smooth regular external surface. Hence, distortions were less, depth was well appreciated, and the instruments manipulation reported to be easier through all the stages and the operations were non-complicated. However, these lenses are ineffective in case of severe central corneal scarring, because RGP lenses are designed to overcome the irregular astigmatism rather than the corneal opacities [14].

## CONCLUSION

This was the first report of a large modified custom-made RGP contact lens use in cataract surgery in irregular corneas, which provides optimal surgical milieu and allows any surgeon to perform the operation according to his or her preferences.
